# Humpback Whale Song and Foraging Behavior on an Antarctic Feeding Ground

**DOI:** 10.1371/journal.pone.0051214

**Published:** 2012-12-19

**Authors:** Alison K. Stimpert, Lindsey E. Peavey, Ari S. Friedlaender, Douglas P. Nowacek

**Affiliations:** 1 Department of Oceanography, Naval Postgraduate School, Monterey, California, United States of America; 2 Bren School of Environmental Science & Management, University of California, Santa Barbara, California, United States of America; 3 Nicholas School of the Environment, Duke University Marine Lab, Beaufort, North Carolina, United States of America; 4 Nicholas School of the Environment and Pratt School of Engineering, Duke University Marine Lab, Beaufort, North Carolina, United States of America; Utrecht University, The Netherlands

## Abstract

Reports of humpback whale (*Megaptera novaeangliae*) song chorusing occurring outside the breeding grounds are becoming more common, but song structure and underwater behavior of individual singers on feeding grounds and migration routes remain unknown. Here, ten humpback whales in the Western Antarctic Peninsula were tagged in May 2010 with non-invasive, suction-cup attached tags to study foraging ecology and acoustic behavior. Background song was identified on all ten records, but additionally, acoustic records of two whales showed intense and continuous singing, with a level of organization and structure approaching that of typical breeding ground song. The songs, produced either by the tagged animals or close associates, shared phrase types and theme structure with one another, and some song bouts lasted close to an hour. Dive behavior of tagged animals during the time of sound production showed song occurring during periods of active diving, sometimes to depths greater than 100 m. One tag record also contained song in the presence of feeding lunges identified from the behavioral sensors, indicating that mating displays occur in areas worthy of foraging. These data show behavioral flexibility as the humpbacks manage competing needs to continue to feed and to prepare for the breeding season during late fall. This may also signify an ability to engage in breeding activities outside of the traditional, warm water breeding ground locations.

## Introduction

Migratory species frequently exhibit distinct behaviors during different phases of their migratory cycle. For example, many baleen whale species have separate breeding and feeding grounds, and behavior is believed to be largely discrete between the two. In humpback whales (*Megaptera novaeangliae*), one of the best-studied baleen whales, song is primarily produced on the breeding grounds, during the time of the life cycle in which males are competing for mating opportunities with females.

Humpback whale song is well known as one of the most complex acoustic displays in animals. The most basic element of this acoustic display is termed a “unit,” defined as the shortest sound that still seems continuous to the human ear [1]. Units are combined to form phrases, which are repeated to form themes, which in turn are repeated in a predictable pattern to form a song. Songs last anywhere from a few minutes to over twenty minutes, and then are generally repeated continuously to form a song session, which can last for more than 20 hours [1]–[3].

One of the first theories to describe song function suggested the display was an advertisement to attract females, because it is believed that only the males sing [4], [5]. However, female humpbacks rarely approach singing males (see [6] for an exception), so many other theories have emerged, including song as a display directed towards other males [2], [7], [8], as a migratory beacon [9], to synchronize estrus in females [10], and as a form of biosonar [11]. Song also evolves and changes over time, within discrete wintering populations, and to some extent across ocean basins [12]–[14]. Because of these and other varied properties and complexities, the function of humpback song is still actively studied and debated [3], [15], [16].

Another variation to the general descriptions of singing behavior is humpback song occurring outside of the breeding season, showing a plasticity in the behavior that fuels the function debate as well as enriching our understanding of the behavior and its scope. This “off-season” humpback song, including chorusing (used here to mean multiple whales singing at the same time, not necessarily synchronized), has been reported from several feeding areas and migration routes around the world [9], [17]–[21]. Some overlap has also been found between non-song sound production (un-patterned sounds produced throughout the year [22]–[24]) and song units that are recorded on the migration route in eastern Australia [24]. Most reports of off-season song describe opportunistic recordings during the end (fall) and beginning (spring) of the feeding season, which we refer to as the “shoulder season.” An exception is Vu et al. [25], who describe a continuous year-long dataset from a feeding ground in the Northwest Atlantic in which song was recorded in almost every month of the year, but with clear increases during these shoulder season times.

Most of these studies also describe data recorded by remote hydrophones located in the northern hemisphere. While these instruments provide a measure of when humpbacks are present, overlap in chorusing can make it challenging to isolate a complete song produced by one individual to investigate its structure. Also, no data exist that describe the non-acoustic behavior of an individual whale during singing on the feeding grounds. Understanding the behavior and movements of individual whales during these time periods helps inform our interpretation of this off-season song.

In this study we describe the results from two multi-sensor suction-cup tag deployments on humpback whales in an Antarctic feeding ground, the acoustic records of which contain loud song. These data are the first descriptions of a single individual's song structure from the Antarctic or any other feeding ground, and also of the underwater dive behavior of whales during this song production. We describe the structure and organization of the song recorded, compare the song patterns between the two individuals, and discuss whale behavior with respect to potential overlap or switching between sound production and feeding activity in a single location.

## Methods

### Fieldwork

**Figure 1:**
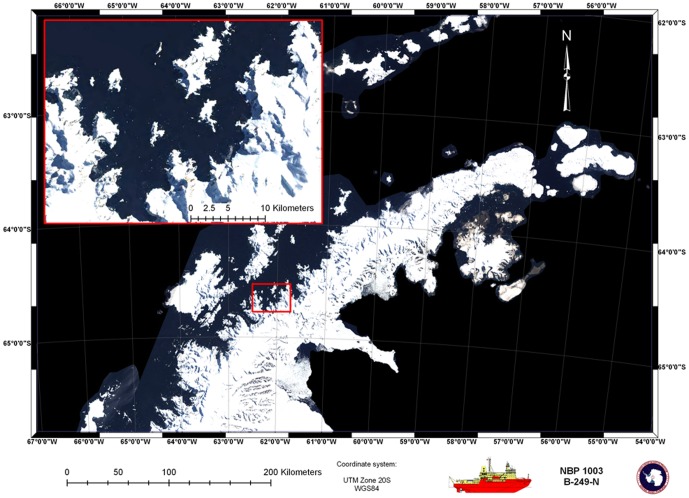
Map of study location off the Western Antarctic Peninsula. Inset shows Wilhelmina Bay, which is east of Anvers Island and Palmer Station. Flandres Bay is two bays south of Wilhelmina, close to Anvers Island. The two focal animals in this study were tagged in Wilhelmina Bay. [Figure by Pat Halpin.]

We tagged ten humpback whales with non-invasive, suction cup acoustic tags (DTAGs [26]) in the waters off of the Western Antarctic Peninsula in the austral fall, between 12 May and 04 June 2010. Research locations included Wilhelmina Bay and Flandres Bay, which are northeast and southeast of Palmer Station, respectively (Figure 1). We worked from the United States Antarctic Program's *RVIB Nathaniel B Palmer*.

Tags were deployed for 24-hour periods, and during that time they continuously recorded acoustics (64 kHz sampling rate) as well as behavioral data from a suite of temperature, pressure, accelerometer, and magnetometer sensors (50 Hz sampling rate, decimated to 5 Hz for analysis). During daylight hours, tagged whales were visually tracked from small, rigid-hulled inflatable boats (RHIBs) conducting focal-individual follows [27], and from the *Palmer* at night via a VHF radio signal from the DTAG.

### Acoustic analysis

Acoustic records were visually scanned for periods of song by two experienced acoustic analysts using the eXtensible Bioacoustic Analysis Tool (XBAT) [28], running in Matlab 7.0. Periods of song (“bouts”) were identified based on high signal-to-noise ratio (SNR) and an obvious pattern in the order in which units were repeated. Designation was subjective, but at minimum, two different themes and repetition of phrases needed to be present before the section could be declared a song bout. Within-bout song units were then individually identified (manually delineated using XBAT), and root-mean-square (RMS) received levels (RLs) were calculated over the full bandwidth of the recording after the frequencies below 400 Hz were emphasized to compensate for the tag hardware's built-in high pass filter. The low frequency emphasis filter combined a high pass filter at 40 Hz and a low pass filter at 400 Hz, resulting in a gain of 20 dB between 40 and 400 Hz [22], [29]. Source levels for these sounds cannot be calculated using this type of data because of the placement of the hydrophone on the animal's body. Its location on the back of the animal, roughly 3 m caudal to the blowholes and approximately on the animal's dorsal midline, is likely behind the sound source and could be in the near-field of any sounds produced by the tagged animal. In addition, some unknown amount of attenuation and distortion may be occurring as the sound propagates through the animal's body and surrounding waters [30].

### Behavioral analysis

It is difficult to definitively ascribe calls recorded on a tag to the focal animal because sounds produced by another animal in close proximity could also appear as intense sounds on the tagged animal's acoustic record [30]. Because these tags had only one hydrophone, and humpbacks habitually travel in closely spaced groups, we could not identify focal calls based on a consistent angle of arrival of successive sounds, or based on a lack of nearby conspecifics [30]–[33]. However, it is likely that if an associate whale were producing song registering strongly on the tag, this whale would be in close enough proximity to be engaged in similar behaviors to the tagged animal [31]. Therefore, we assumed that our broad assessments of dive behavior and suitability of the area for foraging would also apply to other close associates in the group, in the case that some or all of the sounds were produced by a companion.

Sound production was related to behavior by integrating the individual song units with the kinematics record based on time. Periods of song were overlaid on the dive profile to compare timing of song production with tagged animal depth and dive behavior. In addition, the locations of presumed foraging lunges were detected automatically using the algorithm described in Ware et al. [34]. This technique identified vertical lunges based on a significant change in upward or downward acceleration, and all other lunges based on changes in the speed profile calculated using acoustic flow noise as a proxy for speed [35], [36]. Lunges are believed to imply directed feeding on layers of krill in the area [34], [37].

All field research was permitted under the U.S. Marine Mammal Protection Act by the National Marine Fisheries Service Permit 808–1735, the Antarctic Conservation Act Permit 2009-014, and Duke University Institutional Animal Care and Use Permit A041-09-02.

## Results

Background song was evident in the acoustic record of all ten tagged whales. In addition, the records of two whales (“mn132a” and “mn133a”) contained loud and clear song from an individual, for substantial lengths of time. Both of these whales were part of a closely associated pair when first tagged, and later on, whale mn132a was in a group of three animals during the first two bouts of song. We were not able to track the whales visually after sunset (∼1530 local time), so we do not know how many other animals, if any, were in close proximity during later bouts of sound production. Both of the tagged animals remained in Wilhelmina Bay (Figure 1) and song was recorded intermittently throughout the course of the 24-hour tag attachment period. Each record contained multiple song bouts, including a bout close to or slightly over an hour in duration in each case (Table 1).

**Table 1:** Song production on acoustic records of whales mn132a and mn133a.

				Phrase percent occurrence								
Tag acoustic recording	Number of bouts	Range of bout duration	Total duration of singing	BB	PT	LH	LM	LS	UNID	T	P	Total number of phrases
mn132a	8	1 to 50 minutes	119 minutes	8.7	4.6	11.7	11.5	21.0	27.5	10.2	4.8	461
	Number of repetitions/theme (standard deviation)			1 (0.5)	1 (0.3)	2 (2.3)	2 (1.6)	4 (4.0)				
mn133a	4	1 to 68 minutes	115 minutes	9.7	15.0	19.1	19.4	19.7	6.9	7.8	2.2	319
	Number of repetitions/theme (standard deviation)			1 (0.4)	2 (0.9)	2 (0.8)	2 (0.7)	2 (0.8)				

The second half of the table contains percent occurrence of each of the major phrase types, as well as unidentified phrases (UNID), transition phrases (a combination of two neighboring phrases; T), and partial phrases (P). The second line of each record contains the average (and standard deviation) number of repetitions of phrases within a theme.

Received levels of song units, calculated at the tag's location on the animal's back and over the full recording bandwidth, ranged between 111 and 159 dB re 1 µPa RMS (mean 139 dB re 1 µPa over 2811 units for mn132a and mean 138 dB re 1 µPa over 1874 units for mn133a). We did not observe any obvious association between RL and depth, time of day, or neighboring unit RLs (adjacent in time within the song).

### Song structure

**Figure 2:**
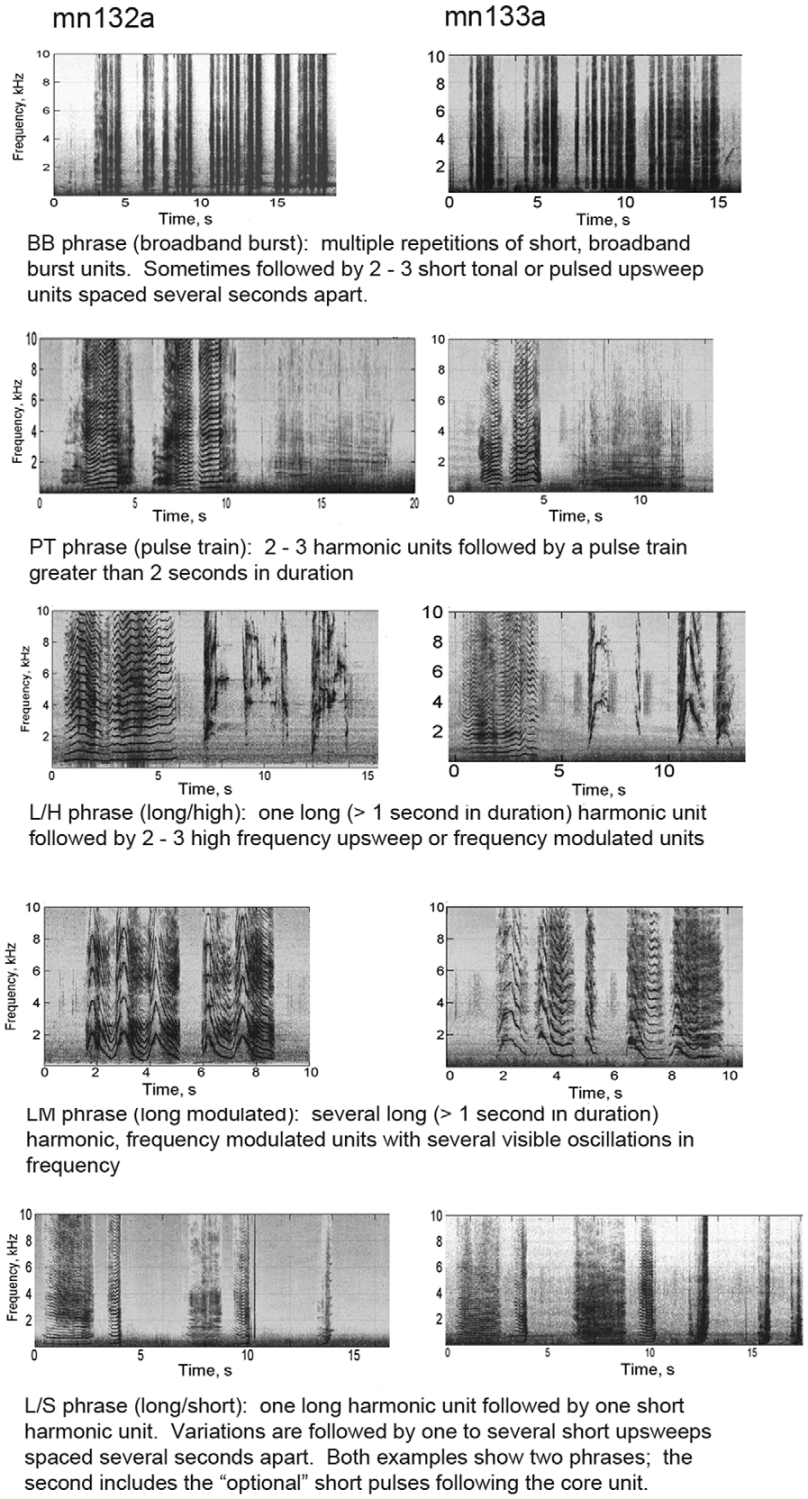
Examples of the most common phrases for each recording. Spectrograms were generated in Matlab (Hamming window, FFT size 2048, 50% overlap).

We named units based on their acoustic properties, using terms such as broadband burst (BB), pulsed (P), long (>1 second, L), short (<1 second, S), and high (>2 kHz, H). For example, alternating long and short units would be labeled “L/S”. The two whales produced similar phrases, and shared themes and overall structure, including five identifiable and organized themes (Figure 2). The L/S theme was the most common and had the highest number of phrase repetitions. Percent occurrence of phrase types and mean number of phrase repetitions within themes are shown in Table 1.

**Figure 3:**
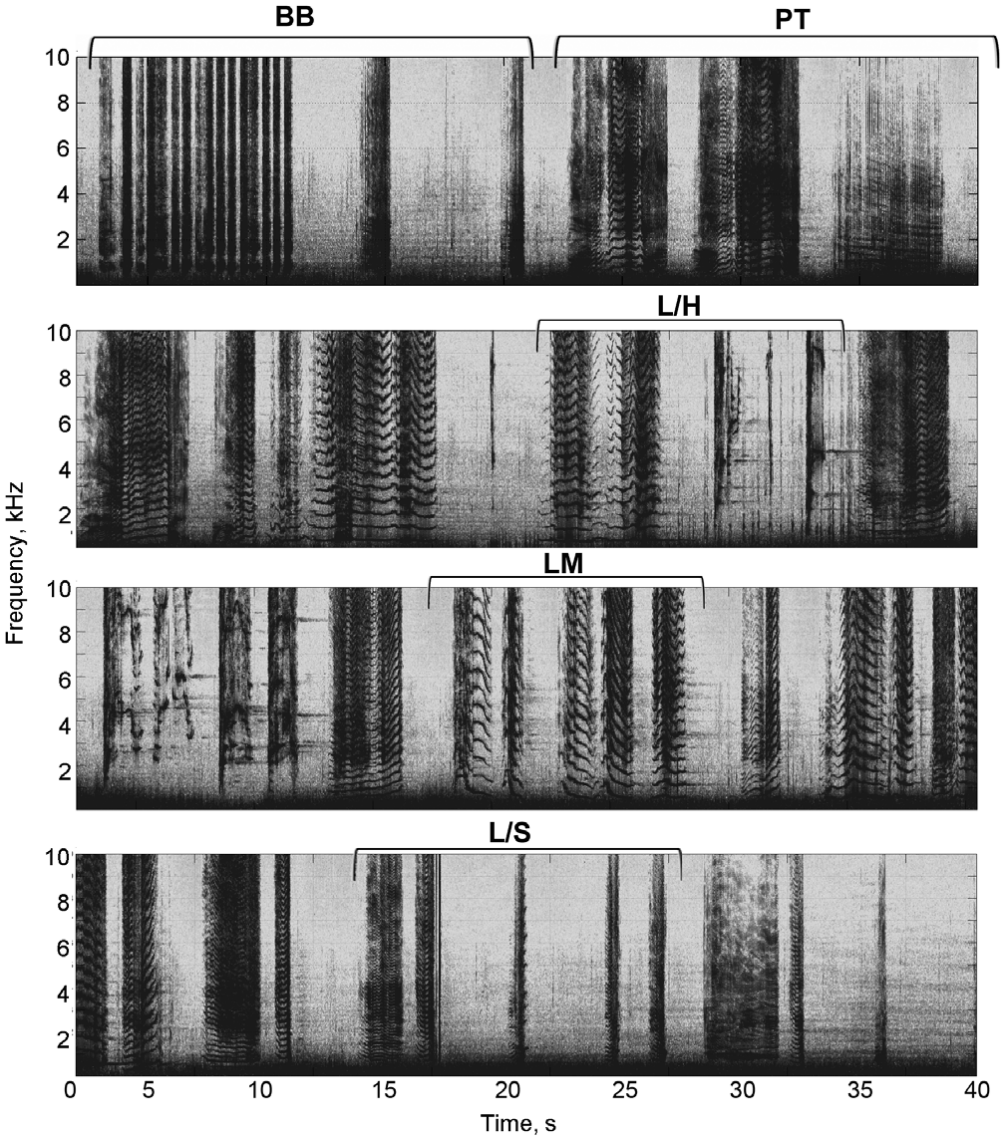
Example section of song from mn132a's acoustic record showing the common structure. Phrases are further described in Figure 2 and Table 1 (BB: broadband bursts; PT: pulse train; L/H: long/high; LM: long modulated; L/S: long/short). Spectrograms were generated in Matlab (Hamming window, FFT size 2048, 50% overlap).

General structure on both records adhered roughly to the following pattern of themes: BB – PT – L/H – LM – L/S. Figure 3 shows an example of this pattern. The songs were not rigidly structured and sometimes not continuous (in some cases up to 28% unidentified phrases, Table 1), but this overall pattern of themes persisted.

### Behavior during song production

**Figure 4:**
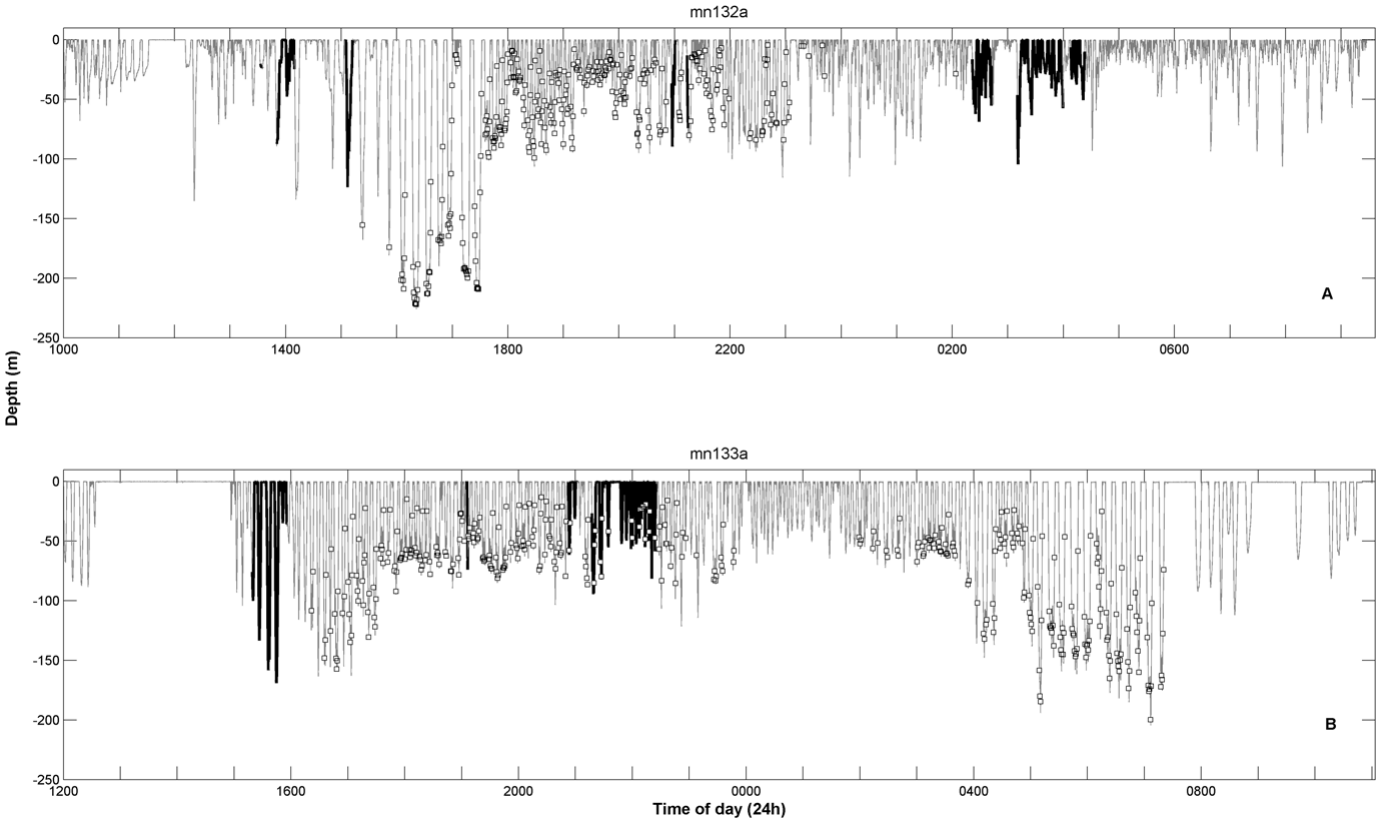
Dive profiles with periods of song marked for mn132a (a) and mn133a (b). Thick black lines denote periods during which song was recorded. Boxes represent automatically detected (and manually validated) locations of lunges.

One of the unique aspects of this dataset is the ability to examine the behavior of animals under water during the song recording. Figure 4 shows the dive profile of each whale over the duration of the tag attachment. Overlaid are locations of feeding lunges that were automatically detected via the acoustic record and verified by an experienced technician who cross-referenced the accelerometer data [34]. Whale mn132a's record had defined periods of feeding that did not overlap with the defined periods of song (the thick black lines in Figure 4). Conversely, whale mn133a's record had two bouts of song during dives that included feeding lunges. This foraging behavior appears to have begun before singing started, and continued well after singing ceased.

## Discussion

These results confirm that humpback whales commonly sing on the feeding grounds in the Western Antarctic Peninsula during late fall, as all tag records contained at least minimal background chorusing. Deployment of DTAGs allowed us to analyze song structure at the level of an individual, something that has not been addressed with remotely collected data documenting song chorusing. Through these tag data we also gain a unique perspective in terms of the underwater diving and movement behavior of whales during times of song production.

### Comparison to traditional song

Given that song is less prevalent on the feeding grounds, the level of structure in the song we recorded was higher than expected. We found a clear pattern of themes sung in a specific order, which is characteristic of humpback whale song as first described by Payne and McVay [1], and this pattern held for both of the whales we recorded singing in this area. Similar phrases were evident in the background chorusing, though theme structure was more difficult to analyze in detail. The type of song units produced was consistent between the two songs as well.

However, our recordings of off-season, or perhaps more accurately, shoulder-season song on this Antarctic feeding ground were not as continuous as song on the breeding grounds during the breeding season, where singers often sing for hours at a time [3]. The two tagged animals' acoustic records here each contained one long bout of song during the 24 hour study period, but also had many shorter sections, which may be similar to the partial song or song fragments mentioned in early reports of humpback whale song recorded on feeding grounds [19], [21]. There were also interspersed unidentified phrases, periods of song that were not very refined (i.e. had no obvious pattern), and periods of non-song sound production.

We do not know for certain the breeding ground(s) of this population of humpback whales. Evidence of linkage between the Western Antarctic Peninsula humpbacks and the breeding ground off the coast of Bahia, Brazil has been gathered via satellite tracking [38]. However, humpbacks travel great distances, as inferred from evidence of acoustic interaction between populations on either side of the Atlantic [39], horizontal cultural transmission of song across the Pacific [14], and fluke matches showing movement of an individual between Brazil and Madagascar [40]. In fact, recent evidence based on fluke matching does show migration between this Western Antarctic feeding ground and a breeding area in American Samoa [41]. Thus, comparisons of this song with that from established breeding grounds will need to be geographically extensive, and will be an enlightening topic of further study. We have included acoustic example files of song units with this paper to facilitate future comparisons (see acoustic files S1 and S2).

### Behavior during song production

Many of the focal whales observed during this research exhibited frequent periods of social activity in addition to feeding, including the two animals discussed here. Song production in a group of three adults (as was the case during the first two bouts of song production on the record of mn132a) is unusual in comparison to the breeding grounds and migration routes, where solitary singers or singers escorting mother/calf pairs are the norm [5], [15], [42]. Another key finding with this work is that the acoustic records of both tagged whales showed song production during periods of active diving, in some cases to depths greater than 100 m. This also differs from the typical singer behavior on the breeding grounds, where whales will frequently sing while remaining stationary at depths between 15 and 25 m [43]. Tagged blue whales also generally sing when at depths shallower than 35 m [32]. Sound production in diving cetaceans is complicated by changing ambient pressure due to depth changes, so these data showing that humpbacks are capable of song production at deeper depths may inform the search to describe the mechanism of sound production in baleen whales [44].

The close overlap between singing and feeding in the record of whale mn133a is surprising. As noted above, it is possible that the song during this portion of the record is actually produced by a companion whale in close proximity. If this were the case, the two individuals would have had to maintain a relatively close fixed-distance association for long periods in order for the song to appear to have been from one animal continuously, or the song levels on the tag may have faded out completely as the other (potentially singing) whale drifted away. As described in the results section, unit received levels did vary over a range greater than 40 dB. However, these levels did not fluctuate in a regular way, i.e. increasing steadily as an associate drifted closer or decreasing steadily as an associate drifted away. Humpback whales are in fact known to vary their source levels during song production. Au and colleagues documented fluctuations of 10 dB re 1 µPa RMS within a given unit type from a single individual, and 22 dB re 1 µPa RMS of variation overall across three individuals and several unit types, as measured using a vertical array with stationary animals [43]. Overall, measurements of sound levels from hydrophones that are possibly in the near field and on an animal's back (thus subject to shading by the body and varying amounts of flow noise from body movements), and that are measuring sounds that may already be fluctuating in intensity, are not a reliable method of identifying the location or identity of a sound-producing baleen whale.

Regardless, even if it were a companion whale singing, whale pairs often engage in similar behaviors when associated [45]–[47], so if one were feeding, its associate may have been as well. At minimum, for the levels to register this strongly on the tag, the companion would have to have been in close enough proximity that the dive depths would be similar between the two individuals, and both animals would have been immersed in the same prey patch.

Thus, this work highlights the issue of tradeoff between foraging and breeding/display behavior while still on the feeding grounds. Both animals appear to have switched between foraging and breeding/display behavior over the course of a 24-hour period, with even a conservative interpretation indicating that groups of animals may have some individuals feeding while others actively engage in mating displays. These results combined with the increasing number of reports of chorusing recorded on humpback feeding grounds [17], [19], [25], competition on the feeding grounds [48], and even occasional reports of feeding on the breeding grounds [49] indicate that “feeding” and “breeding” behavior may be more plastic both spatially and temporally than traditionally thought.

Given the large amount of food available [50], these whales do not necessarily need to feed continuously, which provokes the interesting question of when they would switch from display behavior to foraging and back. The concept of behavioral flexibility has been described as the ability to modify behavior adaptively based on surrounding conditions [51], and humpback whales have shown this ability by developing and learning new foraging behaviors in response to prey type and availability [52]. The whales studied in this Antarctic location showed similar flexibility, exhibiting both display and foraging behavior, and even singing amidst lunge-worthy prey patches.

Humpback whale behavior may be more tied to the time of year than to physical location, in which case this behavioral tradeoff between feeding and mating behaviors would be a common dilemma faced by whales remaining on the feeding grounds later in the year due to varying environmental conditions. Recent work on fin whale song patterns in the Arctic has shown fin whale presence much later into the year than previously thought [53]. The authors suggested that with changing sea ice conditions affecting whale distributions, the behavioral dichotomy of breeding vs. feeding behavior for this migratory species is too simplistic, and mating may be taking place at higher latitudes. Major changes in the extent and duration of sea ice cover around the Antarctic Peninsula [54] may similarly be providing conditions for increases in breeding behavior on the feeding grounds in humpback whales, meriting further study.

## Supporting Information

Acoustic File S1 **Representative song phrases (.wav).** Example phrases for each of the two songs (Acoustic file S1 = record of mn132a and Acoustic File S2 = record of mn133a) are contained in wav files within the supporting information. Representative phrases were selected based on lack of background interference, and are not continuous because phrases were often repeated. Shorter versions of phrases were chosen, and files were decimated to a sampling rate of 44100 Hz in order to comply with the journal's size limits for supporting information. Frequencies below 400 Hz were emphasized to compensate for the tag hardware's built-in high pass filter (see Methods text). In some cases, acoustic sonar pulses may be audible in the background of clips – this is due to the research vessel leaving echosounders on for safety in uncharted waters.
(WAV)

Acoustic File S2 **Representative song phrases (.wav).** Example phrases for each of the two songs (Acoustic file S1 = record of mn132a and Acoustic File S2 = record of mn133a) are contained in wav files within the supporting information. Representative phrases were selected based on lack of background interference, and are not continuous because phrases were often repeated. Shorter versions of phrases were chosen, and files were decimated to a sampling rate of 44100 Hz in order to comply with the journal's size limits for supporting information. Frequencies below 400 Hz were emphasized to compensate for the tag hardware's built-in high pass filter (see Methods text). In some cases, acoustic sonar pulses may be audible in the background of clips – this is due to the research vessel leaving echosounders on for safety in uncharted waters.
(WAV)

## References

[1] Payne RS, McVay S (1971) Songs of humpback whales. Science 173: 585–597. 17833100 10.1126/science.173.3997.585

[2] Darling JD, Jones ME, Nicklin CP (2006) Humpback whale songs: Do they organize males during the breeding season? Behaviour 143: 1051–1101.

[3] Schneider JN (2010) Song structure and spatial dynamics of humpback whales (*Megaptera novaeangliae*) on the breeding grounds. Doctoral dissertation, University of Buffalo: 193 p.

[4] Tyack P (1981) Interactions between singing Hawaiian humpback whales and conspecifics nearby. Behavioral Ecology and Sociobiology 8: 105–116.

[5] Winn HE, Winn LK (1978) The song of the humpback whale, *Megaptera novaeangliae*, in the West Indies. Marine Biology 47: 97–114.

[6] Medrano L, Salinas M, Salas I, Deguevara PL, Aguayo A, et al (1994) Sex Identification of Humpback Whales, *Megaptera novaeangliae*, on the Wintering Grounds of the Mexican Pacific-Ocean. Canadian Journal of Zoology 72: 1771–1774.

[7] Frankel AS, Clark CW, Herman LM, Gabriele CM (1995) Spatial distribution, habitat utilization, and social interactions of humpback whales, *Megaptera novaeangliae*, off Hawaii, determined using acoustic and visual techniques. Canadian Journal of Zoology 73: 1134–1146.

[8] Darling JD, Berube M (2001) Interactions of singing humpback whales with other males. Marine Mammal Science 17: 570–584.

[9] Clapham PJ, Mattila DK (1990) Humpback whale songs as indicators of migration routes. Marine Mammal Science 6: 155–160.

[10] Baker CS, Herman LM (1984) Aggressive behavior between humpback whales (*Megaptera novaeangliae*) wintering in Hawaiian waters. Canadian Journal of Zoology 62: 1922–1937.

[11] Frazer LN, Mercado E (2000) A sonar model for humpback whale song. IEEE Journal of Oceanic Engineering 25: 160–182.

[12] Cerchio S, Jacobsen JK, Norris TF (2001) Temporal and geographical variation in songs of humpback whales, *Megaptera novaeangliae*: synchronous change in Hawaiian and Mexican breeding assemblages. Animal Behaviour 62: 313–329.

[13] Noad MJ, Cato DH, Bryden MM, Jenner MN, Jenner KCS (2000) Cultural revolution in whale songs. Nature 408: 537–537. 10.1038/35046199 11117730

[14] Garland EC, Goldizen AW, Rekdahl ML, Constantine R, Garrigue C, et al (2011) Dynamic horizontal cultural transmission of humpback whale song at the ocean basin scale. Current Biology 21: 687–691. 21497089 10.1016/j.cub.2011.03.019

[15] Smith JN, Goldizen AW, Dunlop RA, Noad MJ (2008) Songs of male humpback whales, *Megaptera novaeangliae*, are involved in intersexual interactions. Animal Behaviour 76: 467–477.

[16] Cholewiak DM (2008) Evaluating the role of song in the humpback whale (*Megaptera novaeangliae*) breeding system with respect to intra-sexual interactions. Doctoral dissertation, Cornell University: 159 p.

[17] Clark CW, Clapham PJ (2004) Acoustic monitoring on a humpback whale (*Megaptera novaeangliae*) feeding ground shows continual singing into late spring. Proceedings of the Royal Society of London Series B-Biological Sciences 271: 1051–1057. 10.1098/rspb.2004.2699 PMC1691688 15293859

[18] Charif RA, Clapham PJ, Clark CW (2001) Acoustic detections of singing humpback whales in deep waters off the British Isles. Marine Mammal Science 17: 751–768.

[19] Mattila D, Guinee LN, Mayo CA (1987) Humpback whale songs on a North Atlantic Feeding Ground. Journal of Mammalogy 68: 880–883.

[20] Norris TF, Mc Donald M, Barlow J (1999) Acoustic detections of singing humpback whales (*Megaptera novaeangliae*) in the eastern North Pacific during their northbound migration. Journal of the Acoustical Society of America 106: 506–514. 10420640 10.1121/1.427071

[21] McSweeney D, Chu K, Dolphin WF, Guinee LN (1989) North Pacific humpback whale songs: A comparison of southeast Alaskan feeding ground songs with Hawaiian wintering ground songs. Marine Mammal Science 5: 139–148.

[22] Stimpert AK, Au WWL, Parks SE, Hurst T, Wiley DN (2011) Common humpback whale (*Megaptera novaeangliae*) sound types for passive acoustic monitoring. Journal of the Acoustical Society of America 129: 476–482. 21303027 10.1121/1.3504708

[23] Stimpert AK (2010) Non-song sound production and its behavioral context in humpback whales (Megaptera novaeangliae). Doctoral dissertation, University of Hawaii at Manoa 117 p.

[24] Dunlop RA, Noad MJ, Cato DH, Stokes D (2007) The social vocalization repertoire of east Australian migrating humpback whales (*Megaptera novaeangliae*). Journal of the Acoustical Society of America 122: 2893–2905. 18189579 10.1121/1.2783115

[25] Vu ET, Risch D, Clark C, Gaylord S, Hatch L, et al (2012) Humpback whale song occurs extensively on feeding grounds in the western North Atlantic Ocean. Aquatic Biology 14: 175–183.

[26] Johnson MP, Tyack PL (2003) A digital acoustic recording tag for measuring the response of wild marine mammals to sound. IEEE Journal of Oceanic Engineering 28: 3–12.

[27] Altmann J (1974) Observational Study of Behavior: Sampling Methods. Behaviour 49: 227–267. 4597405 10.1163/156853974x00534

[28] Mills HG, Figueroa HK (2005) Extensible bioacoustical analysis software: Two examples (Abstract). Journal of the Acoustical Society of America 117: 2525.

[29] Aguilar-Soto N, Johnson MJ, Madsen PT, Tyack P, Bocconcelli A, et al (2006) Does intense ship noise disrupt foraging in deep-diving Cuvier's beaked whales (*Ziphius cavirostris*)? Marine Mammal Science 22: 690–699.

[30] Johnson M, Aguilar de Soto N, Madsen PT (2009) Studying the behaviour and sensory ecology of marine mammals using acoustic recording tags: a review. Marine Ecology Progress Series 395: 55–73.

[31] Jensen FH, Bejder L, Wahlberg M, Soto NA, Johnson M, et al (2009) Vessel noise effects on delphinid communication. Marine Ecology Progress Series 395: 161–175.

[32] Oleson EM, Calambokidis J, Burgess WC, McDonald MA, LeDuc CA, et al (2007) Behavioral context of call production by eastern North Pacific blue whales. Marine Ecology Progress Series 330: 269–284.

[33] Parks SE, Searby A, Celerier A, Johnson M, Nowacek DP, et al (2011) Sound production behavior of individual North Atlantic right whales: implications for passive acoustic monitoring. Endangered Species Research 15: 63–76.

[34] Ware C, Friedlaender AS, Nowacek DP (2011) Shallow and deep lunge feeding of humpback whales in fjords of the West Antarctic Peninsula. Marine Mammal Science 27: 587–605.

[35] Goldbogen JA, Calambokidis J, Shadwick RE, Oleson EM, McDonald MA, et al (2006) Kinematics of foraging dives and lunge-feeding in fin whales. Journal of Experimental Biology 209: 1231–1244. 16547295 10.1242/jeb.02135

[36] Simon M, Johnson M, Madsen PT (2012) Keeping momentum with a mouthful of water: behavior and kinematics of humpback whale lunge feeding. Journal of Experimental Biology 215: 3786–3798. 23053368 10.1242/jeb.071092

[37] Tyson RB, Friedlaender AS, Ware C, Stimpert AK, Nowacek DP (2012) Synchronous mother and calf foraging behaviour in humpback whales (*Megaptera novaeangliae*): insights from multi-sensor suction cup tags. Marine Ecology Progress Series 457: 209–220.

[38] Zerbini AN, Andriolo A, Heide-Jorgensen MP, Pizzorno JL, Maia YG, et al (2006) Satellite-monitored movements of humpback whales (*Megaptera novaeangliae*) in the southwest Atlantic Ocean. Marine Ecology Progress Series 313: 295–304.

[39] Darling JD, Sousa-Lima RS (2005) Songs indicate interaction between humpback whale (*Megaptera novaeangliae*) populations in the western and eastern South Atlantic Ocean. Marine Mammal Science 21: 557–566.

[40] Stevick P, Neves MC, Johansen F, Engel MH, Allen J, et al (2011) A quarter of a world away: female humpback whale moves 10000 km between breeding areas. Biology Letters 7: 299–302. 20943678 10.1098/rsbl.2010.0717 PMC3061163

[41] Robbins J, Dalla Rosa L, Allen JM, Mattila D, Secchi E, et al (2011) Return movement of a humpback whale between the Antarctic Peninsula and American Samoa: a seasonal migration record. Endangered Species Research 13: 117–121.

[42] Helweg DA, Frankel A, Mobley J, Herman LM (1992) Humpback whale song: Our current understanding. In: Thomas JA, Kastelein R, Supin AY, editors. Marine Mammal Sensory Systems. New York, NY: Plenum, Inc. pp. 459–483.

[43] Au WWL, Pack AA, Lammers MO, Herman LM, Deakos MH, et al (2006) Acoustic properties of humpback whale songs. Journal of the Acoustical Society of America 120: 1103–1110. 16938996 10.1121/1.2211547

[44] Reidenberg JS, Laitman JT (2007) Discovery of a low frequency sound source in mysticeti (Baleen whales): Anatomical establishment of a vocal fold homolog. Anatomical Record-Advances in Integrative Anatomy and Evolutionary Biology 290: 745–759. 10.1002/ar.20544 17516447

[45] Helweg DA, Herman LM (1994) Diurnal patterns of behavior and group membership of humpback whales (*Megaptera novaeangliae*) wintering in Hawaiian waters. Ethology 98: 298–311.

[46] Zimmer WMX, Johnson MP, Madsen PT, Tyack PL (2005) Echolocation clicks of free-ranging Cuvier's beaked whales (*Ziphius cavirostris*). Journal of the Acoustical Society of America 117: 3919–3927. 16018493 10.1121/1.1910225

[47] Whitehead H (1983) Structure and stability of humpback whale groups off Newfoundland. Canadian Journal of Zoology 61: 1391–1397.

[48] Weinrich M (1995) Humpback whale competitive groups observed on a high latitude feeding ground. Marine Mammal Science 11: 251–254.

[49] Alves LCPD, Andriolo A, Zerbini AN, Pizzorno JLA, Clapham PJ (2009) Record of feeding by humpback whales (*Megaptera novaeangliae*) in tropical waters off Brazil. Marine Mammal Science 25: 416–419.

[50] Nowacek DP, Friedlaender AS, Halpin PN, Hazen EL, Johnston DW, et al (2011) Super-aggregations of krill and humpback whales in Wilhelmina Bay, Antarctic Peninsula. PLoS One 6: e19173 doi:19110.11371/journal.pone.0019173. 21556153 10.1371/journal.pone.0019173 PMC3083408

[51] Wright TF, Eberhard JR, Hobson EA, Avery ML, Russello MA (2010) Behavioral flexibility and species invasions: the adaptive flexibility hypothesis. Ethology Ecology and Evolution 22: 393–404.

[52] Weinrich MT, Schilling MR, Belt CR (1992) Evidence for acquisition of a novel feeding behavior - lobtail feeding in humpback whales, *Megaptera novaeangliae* . Animal Behaviour 44: 1059–1072.

[53] Simon M, Stafford KM, Beedholm K, Lee CM, Madsen PT (2010) Singing behavior of fin whales in the Davis Strait with implications for mating, migration and foraging. Journal of the Acoustical Society of America 128: 3200–3210. 21110615 10.1121/1.3495946

[54] Stammerjohn SE, Martinson DG, Smith RC, Iannuzzi RA (2008) Sea ice in the western Antarctic Peninsula region: Spatio-temporal variability from ecological and climate change perspectives. Deep-Sea Research II 55: 2041–2058.

